# Toxicity of overexpressed MeCP2 is independent of HDAC3 activity

**DOI:** 10.1101/gad.320325.118

**Published:** 2018-12-01

**Authors:** Martha V. Koerner, Laura FitzPatrick, Jim Selfridge, Jacky Guy, Dina De Sousa, Rebekah Tillotson, Alastair Kerr, Zheng Sun, Mitchell A. Lazar, Matthew J. Lyst, Adrian Bird

**Affiliations:** 1 Wellcome Centre for Cell Biology, University of Edinburgh, Edinburgh EH9 3BF, United Kingdom;; 2 Institute for Diabetes, Obesity, and Metabolism, University of Pennsylvania Perelman School of Medicine, Philadelphia, Pennsylvania 19104, USA

**Keywords:** histone deacetylase 3, MeCP2, NCoR

## Abstract

Duplication of the X-linked *MECP2* gene causes a severe neurological syndrome whose molecular basis is poorly understood. To determine the contribution of known functional domains to overexpression toxicity, we engineered a mouse model that expresses wild-type or mutated MeCP2 from the *Mapt* (*Tau*) locus in addition to the endogenous protein. Animals that expressed approximately four times the wild-type level of MeCP2 failed to survive to weaning. Strikingly, a single amino acid substitution that prevents MeCP2 from binding to the TBL1X(R1) subunit of nuclear receptor corepressor 1/2 (NCoR1/2) complexes, when expressed at equivalent high levels, was phenotypically indistinguishable from wild type, suggesting that excessive corepressor recruitment underlies toxicity. In contrast, mutations affecting the DNA-binding domain were toxic when overexpressed. As the NCoR1/2 corepressors are thought to act through histone deacetylation by histone deacetylase 3 (HDAC3), we asked whether mutations in NCoR1 and NCoR2 that drastically reduced their ability to activate this enzyme would relieve the MeCP2 overexpression phenotype. Surprisingly, severity was unaffected, indicating that the catalytic activity of HDAC3 is not the mediator of toxicity. Our findings shed light on the molecular mechanisms underlying *MECP2* duplication syndrome and call for a re-evaluation of the precise biological role played by corepressor recruitment.

## Abstract (precis)

In this study, Koerner et al. investigated the molecular basis of a severe neurological syndrome caused by duplication of the X-linked MECP2 gene. To determine the contribution of known functional domains to overexpression toxicity, they generated a mouse model that expresses wild-type or mutated MeCP2 from the Mapt (Tau) locus in addition to the endogenous protein. Their findings delineate the molecular mechanisms underlying MECP2 duplication syndrome and suggest a re-examination of the biological role played by corepressor recruitment.

Mutations involving the X-linked *MECP2* gene cause an array of human neurological disorders. Mosaicism for severe deficiency of functional MeCP2 protein product causes Rett syndrome, predominantly in females (Amir et al. 1999), whereas excess protein causes *MECP2* duplication syndrome, mostly affecting males (Van Esch et al. 2005). Molecular analysis of Rett syndrome missense mutations, supported by equivalent genetic models in mice, has identified two discrete regions of the protein within which missense mutations are concentrated. These coincide with and inactivate two domains in the MeCP2 protein that were identified in functional assays. The methyl-CpG-binding domain (MBD) binds to methylated sites in DNA (Lewis et al. 1992; Nan et al. 1993), whereas the nuclear receptor corepressor (NCoR) interaction domain (NID) interacts with a specific subunit—TBL1X(R1)—that is shared by the NCoR1/2 corepressor complexes (Lyst et al. 2013; Kruusvee et al. 2017). Accordingly, mice retaining these two domains but lacking the remaining two thirds of the protein are fully viable, with relatively mild phenotypic defects (Tillotson et al. 2017). These and other data are compatible with the view that MeCP2 functions primarily by recruiting the NCoR1/2 corepressors to methylated sites in the genome (Lyst and Bird 2015). This scenario also agrees with evidence that loss of MeCP2 leads to up-regulation of genes in proportion to their MeCP2 occupancy in the wild-type mouse brain (Kinde et al. 2016; Lagger et al. 2017).

As MeCP2 overexpression causes a disorder that is clinically distinct from Rett syndrome, it is important to establish whether toxicity is due to an excess of the same two functional domains that are mutated in Rett syndrome or whether other parts of the protein are involved. A mouse model of overexpression has been created by expressing the human *MECP2* gene integrated as a transgene in addition to the endogenous X-linked gene (Collins et al. 2004). The resulting phenotype recapitulates aspects of the human condition, although the phenotype is relatively mild. Creation of equivalent models using *MECP2* transgenes carrying missense mutations that interfere with either NID or MBD function failed to register deleterious phenotypes, suggesting that these two domains must be intact for overexpression to be detrimental (Heckman et al. 2014). To test this with increased stringency, we used overexpression of the coding region of MeCP2 from the autosomal *Mapt* (*Tau*) locus, which achieves higher MeCP2 abundance and leads to a severe phenotype. Luikenhuis et al. (2004) used this method previously to show that mice lacking the endogenous *Mecp2* gene could be rescued by the Tau-MeCP2 fusion protein expressed heterozygously but that high expression in homozygous animals was severely detrimental. Extending this approach, we found that ∼3.8-fold overexpression of MeCP2 is lethal in mice, but a missense mutation in the NID exogenously expressed at a similarly high level is benign. Our results emphasize that excess NID is an essential mediator of overexpression toxicity. In contrast, less extreme overexpression of hypomorphic MBD mutants that cause Rett syndrome was deleterious, arguing that the enhanced expression of these hypomorphic forms of MeCP2 in the presence of the wild-type protein may not offer therapeutic benefit (Lamonica et al. 2017). As the NID interacts with a shared subunit of the NCoR1/2 corepressors, it seemed plausible that overrecruitment might cause excessive repression mediated by a catalytically active component of these complexes: histone deacetylase 3 (HDAC3) (Guenther et al. 2001). To test this hypothesis genetically, we asked whether toxicity associated with overexpression of MeCP2 in mice could be ameliorated by mutation of the deacetylase activation domains (DADs) of both NCoR1 and NCoR2 (You et al. 2013). Our results do not support the hypothesis, as the phenotypes of mice overexpressing MeCP2 were no less severe when the double-DAD mutation was introduced into these animals. Our results suggest that other aspects of corepressor function, distinct from HDAC3 activity, are deleterious when excessive. Thus, while underlining the importance of the NID as the primary underlying cause of *MECP2* duplication syndrome, the findings raise questions about downstream mechanisms that mediate this effect.

## Results

### MeCP2 is expressed from the Tau locus at wild-type levels in neurons and glia

**Figure 1.**
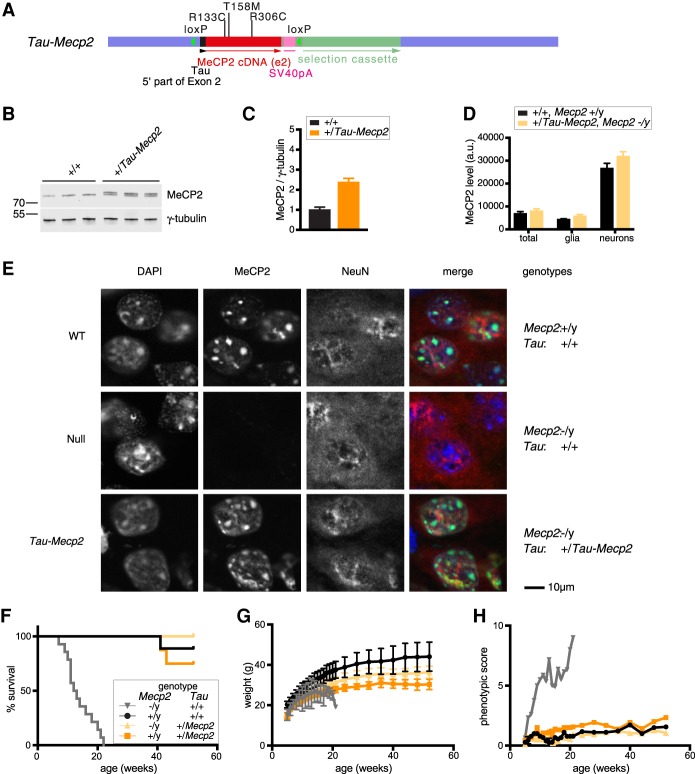
MeCP2 is expressed from the *Tau* locus at wild-type levels in neurons and glia. (*A*) Schematic representation of the *Tau-Mecp2* allele showing the intronic genomic DNA region of the *Tau* (*Mapt*) locus (blue bar), the loxP sites (green triangle), the 5′ extremity of exon 2 of *Tau* (black bar), the MeCP2 isoform e2 cDNA (red bar), the SV40 polyA signal (pink bar), and a selection cassette comprising the *PGK* promoter, neomycin resistance gene, and *PGK* polyA signal (pale-green bar). Location of introduced missense mutations (R133C, T158M, and R306C) within MeCP2 are shown as vertical black lines. (*B*) Western blots of three brains of *+/Tau-Mecp2* and wild-type littermates (males, 7 wk) probed for MeCP2 and γ-tubulin. (*C*) Quantification of *B* (mean and standard deviation). (Black bar) Wild-type; (orange bar) *Tau-MeCP2*. Two-tailed unpaired *t*-test. *n* = 3 for both genotypes. *P* = 0.0005. (*D*) FACS analysis of nuclei expressing Tau-MeCP2 in MeCP2 knockout background (pale orange) and wild-type (black) littermates. The histogram shows MeCP2 fluorescence intensity in the total brain fraction, the NeuN-low fraction (mostly glia), and the NeuN-high fraction (mostly neurons) of nuclei. Shown are the mean and standard deviation of three biological replicates. Two-tailed unpaired *t*-test. *n* = 3 for both genotypes. *P* = 0.1853 total; *P* = 0.0300 glia; *P* = 0.0385 neurons. (*E*) Immunofluorescence of the hippocampus CA3 brain region of male mice (7 wk of age) with the following genotypes: *Mecp2^+^/y, Tau^+/+^* (wild type); *Mecp2^−^/y, Tau^+/+^* (null); and *Mecp2^−/y^, Tau^+^/Tau-MeCP2* (Tau-MeCP2). Sections were stained for DAPI, MeCP2, and NeuN. (*F*) Survival of cohorts with the following genotypes: *Mecp2^−^/y, Tau^+/+^* (gray line; *n* = 14); *Mecp2^+^/y, Tau^+/+^* (black line; *n* = 9); *Mecp2^−^/y, Tau^+^/Tau-Mecp2* (pale-orange line; *n* = 9); and *Mecp2^+^/y, Tau^+^/Tau-Mecp2* (orange line; *n* = 8). Each genotype was compared with wild type using a Mantel-Cox test. *P* = 0.4897 for *Tau^+^/Tau-Mecp2, Mecp2^+^/y*; *P* = 0.3173 for *Tau^+^/Tau-Mecp2, Mecp2^−^/y*; *P* < 0.0001 for *Tau^+/+^, Mecp2^−^/y*. (*G*) Body weight of the mice shown in *F* (mean and standard deviation). As animals were dying during the scoring period, repeated measures of ANOVA covering the entire time course was not possible. (*H*) Phenotypic score of the mice shown in *F*. As animals were dying during the scoring period, repeated measures of ANOVA covering the entire time course was not possible.

In order to create a model of *MECP2* duplication syndrome, we chose to replace the endogenous *Tau*-coding region with an *Mecp2* cDNA using constructs similar to those described previously (Fig. 1A; Luikenhuis et al. 2004). Specifically, we inserted a cDNA encoding the entire e2 isoform of MeCP2 in-frame downstream from the N-terminal 31 codons of the *Tau*-coding region (Supplemental Fig. S1A–D). Western blot assays of MeCP2 in *Tau-Mecp2* heterozygote brains confirmed similar expression levels of Tau-MeCP2 and the slightly shorter wild-type MeCP2 (Fig. 1B,C). Although Tau was initially thought to be a neuron-specific protein, it is also expressed at lower levels in glia (LoPresti et al. 1995). Accordingly, MeCP2 expression derived from the *Tau-Mecp2* recombined locus was detected by flow cytometry of both neuronal (NeuN-high) and glial (NeuN-low) nuclei in mice lacking an endogenous *Mecp2* gene (Fig. 1D). The wild-type pattern of high MeCP2 levels in neurons and lower levels in nonneuronal cells was recapitulated in mouse brains heterozygous for *Tau-Mecp2* on an *Mecp2*^−^/y mouse background, although we noted slightly elevated levels compared with wild-type in both cell types. Immunostaining with an anti-MeCP2 antibody confirmed that MeCP2 signal was abundant in *Tau-Mecp2* neurons (Fig. 1E). We conclude that the expression profile of *Mecp2* from a heterologous autosomal promoter and locus is remarkably similar to that seen from the endogenous locus in wild-type mice. Importantly, we confirmed a previous report (Luikenhuis et al. 2004) that the Rett-like phenotypes observed in *Mecp2*-null mice can be rescued by exogenous expression of MeCP2 from the *Tau* locus (Fig. 1F–H).

### A 2.4-fold overexpression of MeCP2 is well tolerated, but 3.8-fold overexpression is lethal

**Figure 2.**
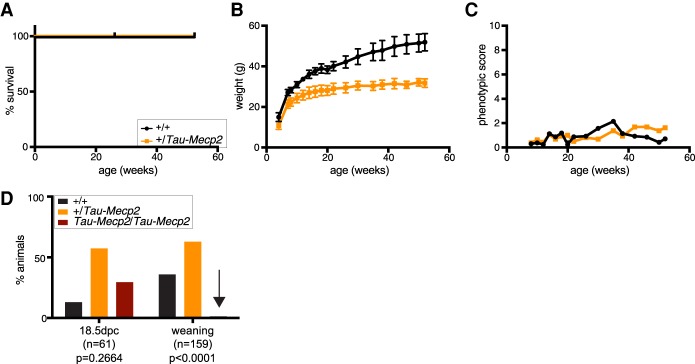
A 2.4-fold overexpression of MeCP2 is well tolerated, but 3.8-fold overexpression is lethal. (*A*) Survival of a cohort comprising the following genotypes: *+/+* (black line; *n* = 8) and *+/Tau-Mecp2* (orange line; *n* = 8). (*B*) Body weight of the mice shown in *A* (mean and standard deviation). Two-way repeated measures of ANOVA over the whole time course but excluding the animal that died were performed. *n* = 7 wild type; *n* = 8 heterozygous. Genotype effect: *F*_(1,13)_ = 102.7. *P* < 0.0001. (*C*) Phenotypic score of the mice shown in *A*. Two-way repeated measures of ANOVA over the whole time course but excluding the animal that died were performed. *n* = 7wild type; *n* = 8 heterozygous. Genotype effect: *F*_(1,13)_ = 0.2809. *P* = 0.6051. (*D*) Genotypes from *Tau-Mecp2* heterozygote intercrossings observed at 18.5 d post-coitum (dpc) and at weaning. Genotypes were *+/+* (black bars), *+/Tau-Mecp2* (orange bars), and *Tau-Mecp2/Tau-Mecp2* (dark-red bars). Although homozyous animals were recovered at a Mendelian ratio at 18.5 dpc, only two homozygous animals were observed at weaning (this is emphasized by the black arrow). One of those had to be culled due to hydrocephalus and severe runting; the second animal survived >1 yr (see Supplemental Fig. S2D,E). A χ^2^ test was used to test for normal Mendelian ratios of genotypes. Embryonic day 18.5 (E18.5): χ^2^= 2.646, *P* = 0.2664; at weaning: χ^2^= 39.58, *P* < 0.0001.

Having established that Tau-MeCP2 is expressed at appropriate levels and can phenotypically rescue *Mecp2*-null mice, we asked whether overexpression gave a phenotype that mimics *MECP2* duplication syndrome. Mice wild type for the endogenous *Mecp2* gene, but additionally expressing one *Tau-Mecp2* allele, expressed, in total, ∼2.4 times wild-type levels of the protein (Fig. 1C). Remarkably, despite significant overexpression, these mice were viable and fertile and survived for >1 yr (Fig. 2A–C). However, they exhibited reduced body weight, and we additionally noted a very late-onset (∼40 wk) breathing phenotype manifest as “panting.” Further analysis using the elevated plus maze, a test for anxiety, showed no difference in time spent in open versus closed arms, although mobility was somewhat reduced in this test (Supplemental Fig. S2A,B). A 3-d learning paradigm on the accelerating rotarod revealed no significant difference compared with wild-type animals (Supplemental Fig. S2C).

In contrast, mice homozygous for the *Tau-Mecp2* allele in the presence of the endogenous *Mecp2* locus, which were anticipated to express 3.8-fold more MeCP2 protein than wild-type (1 + 1.4 + 1.4) were not recovered (Fig. 2D). We expected ∼40 animals with this genotype among 159 progeny, but only two animals reached weaning, one of which had to be culled due to a severely runted phenotype and hydrocephalus. However, analysis of litters at 18.5 d post-coitum (dpc) revealed the expected Mendelian proportions of this genotype, indicating that death occurred at or soon after birth (Fig. 2D). As this genotype results in a *Tau*-null mouse, we wished to confirm that the phenotype was not due to lack of Tau. In line with previous reports (Harada et al. 1994; Dawson et al. 2001), we found that homozygous *Tau*-null mice were phenotypically normal, as weight, survival, and phenotypic score (an aggregate score analyzing general appearance, activity, gait, tremor, breathing, and hindlimb clasping) were indistinguishable from wild-type over 1 yr (Supplemental Fig. S2D–G).

Molecular analysis confirmed increased expression of Tau-MeCP2 in the single surviving animal, as expected (Supplemental Fig. S2H). In line with the reduced weight observed in *Tau-Mecp2* heterozygous animals, the *Tau-Mecp2* homozygous animal had even further decreased weight (Supplemental Fig. S2I). It is notable that *Tau-Mecp2* homozygous mice lacking endogenous MeCP2 expression were recovered at normal Mendelian frequencies. Thus, the combination of Tau deletion and 2.8-fold MeCP2 overexpression does not lead to synthetic lethality, ruling out an interaction between these mutations (Supplemental Fig. S2J). The results indicate that expression of MeCP2 in mice at ∼2.4–2.8 times the wild-type level is unexpectedly benign, but ∼3.8 times the wild-type level is severely toxic. The observed homozygous lethality in our model represented a more severe phenotype than reported previously (Luikenhuis et al. 2004), which may be due to differences in genetic background in this mouse strain or husbandry differences.

### Mutating the NID rescues the lethality caused by MeCP2 overexpression

**Figure 3.**
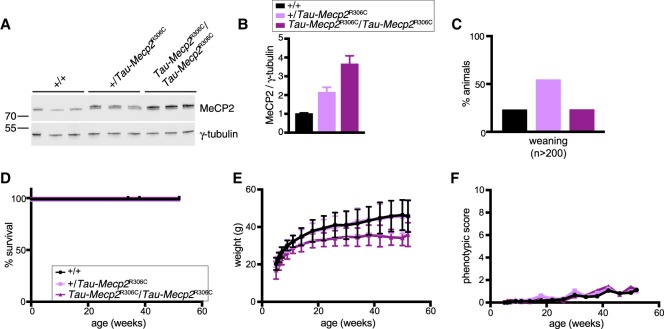
A mutation in the NID renders high MeCP2 overexpression viable. (*A*) Western blots of three brains of animals heterozygous and heterozygous for *Tau-Mecp2[R306C]* and of wild-type littermates (males, 7 wk) probed for MeCP2 and γ-tubulin. (*B*) Quantification of Western blots shown in *A* (mean and standard deviation). (Black) *+/+*; (light purple) *+/Tau-Mecp2[R306C]*; (dark purple) *Tau-Mecp2[R306C]/Tau-Mecp2[R306C]*. Two-tailed unpaired *t*-test. *n* = 3 for each genotype. *P* = 0.0020 for wild type versus heterozygous; *P* = 0.0005 for wild type versus homozygous. (*C*) Genotype distribution from *Tau-Mecp2[R306C]* heterozygous intercrossings observed at weaning. Legend is as for *B*. A χ^2^ test was used to test for normal Mendelian ratios of genotypes. *P* = 0.7258. (*D*) Survival of a cohort comprising *+/+* (*n* = 9), *+/Tau-Mecp2[R306C]* (*n* = 9), and *Tau-Mecp2[R306C]/Tau-Mecp2[R306C]* (*n* = 9). (*E*) Body weights of the mice shown in *D* (mean and standard deviation). Two-way repeated measures of ANOVA (excluding one wild-type and one homozygous animal that died) were performed. *n* = 8 wild type; *n* = 9 heterozygous; *n* = 8 homozygous. Genotype effect: *F*_(2,22)_ = 5.807. *P* = 0.0094. (*F*) Phenotypic score of the mice shown in *D*. Two-way repeated measures of ANOVA (excluding one wild-type and one homozygous animal that died) were performed. *n* = 8 wild type; *n* = 9 heterozygous; *n* = 8 heterozygous. Genotype effect: *F*_(2,22)_ = 0.309. *P* = 0.7373.

We next asked whether mutations that cause Rett syndrome alter toxicity when overexpressed in this system. Initially, the relatively frequent mutation R306C, which disrupts the interaction between MeCP2 and the NCoR1/2 corepressor complexes, was introduced at the *Tau* locus (Supplemental Fig. S3A,B). Heterozygous expression of this allele in mice deficient for endogenous MeCP2 gave rise to typical features of Rett syndrome similar to those observed when the endogenous locus is mutated (Supplemental Fig. S3C–F; Lyst et al. 2013; Brown et al. 2016). We noted that a double dose of the *Tau-Mecp2[R306C]* allele in homozygotes on an *Mecp2*-null background improved survival compared with heterozygotes (Supplemental Fig. S3C). This argues that this mutation retains weak MeCP2 function, in agreement with its somewhat less severe phenotype in mice and humans (Cuddapah et al. 2014; Brown et al. 2016). It is noticeable that an increased dose of this hypomorphic allele improved survival but not phenotypic scoring. Expression of the *Tau-Mecp2[R306C]* allele in a wild-type background raised total MeCP2 to the same total abundance as seen with wild-type *Tau-Mecp2*, reaching ∼3.6-fold in *Tau-Mecp2[R306C]* homozygotes (Fig. 3A,B). Remarkably, in contrast to the neonatal lethal phenotype seen with overexpressed wild-type MeCP2, mice expressing transgenic R306C at the same high level were indistinguishable from wild-type littermates (Fig. 3C–F). In the elevated plus maze, homozygous *Tau-MeCP2[R306C]* mice showed normal avoidance of the open arms but modestly reduced distance traveled during the test (Supplemental Fig. S3G,H). The accelerating rotarod learning paradigm also revealed no significant differences compared with wild-type littermates (Supplemental Fig. S3I). We conclude that this single amino acid substitution effectively abolishes the toxicity caused by MeCP2 overexpression.

### Overexpressing MBD mutants in the presence of wild-type MeCP2 causes neurological defects

**Figure 4.**
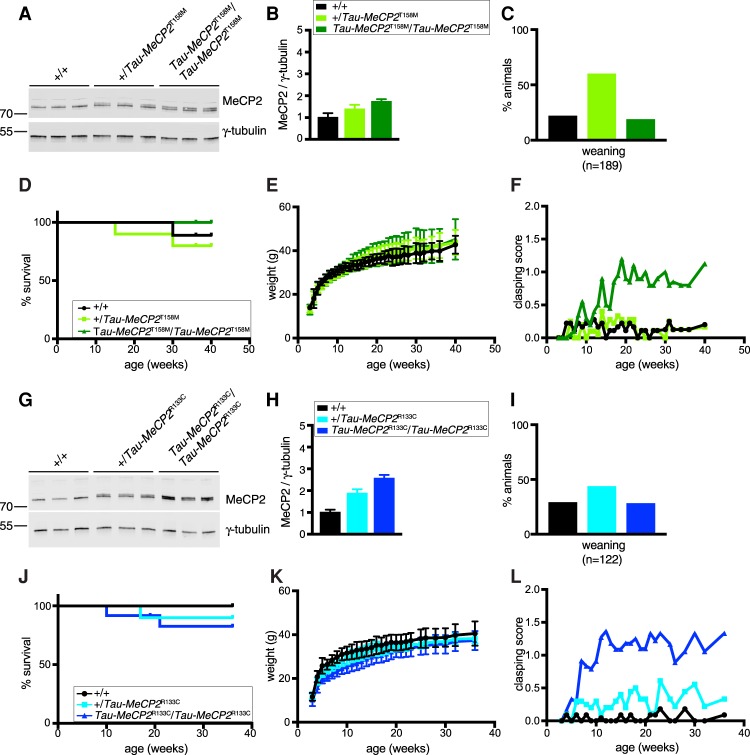
Overexpression of MBD mutants in addition to wild-type MeCP2 is deleterious. (*A*) Western blots of three brains of animals heterozygous and homozygous for *Tau-Mecp2[T158M]* and wild-type littermates (males, 7 wk) probed for MeCP2 and γ-tubulin. (*B*) Quantification of blots shown in *A* (mean and standard deviation). Genotypes are *+/+* (black), *+/Tau-Mecp2[T158M]* (light green), and *Tau-Mecp2[T158M]/Tau-Mecp2[T158M]* (dark green). A two-tailed unpaired *t*-test was used for statistical analysis. *P* = 0.0748 for wild type versus heterozygous; *P* = 0.0053 for wild type versus homozygous. (*C*) Genotypes derived from *Tau-Mecp2[T158M]* heterozygous intercrossings observed at weaning. A χ^2^ test was used to test for normal Mendelian ratios of genotypes. *P* = 0.1554. (*D*) Survival of a cohort comprising *+/+* (black; *n* = 9), *+/Tau-MeCP2[T158M]* (bright green; *n* = 10), and *Tau-Mecp2[T158M]/Tau-Mecp2[T158M]* (dark green; *n* = 10). A Mantel-Cox test was used to analyze survival. *P* = 0.5767 for wild type versus heterozygous; *P* = 0.2918 for wild type versus homozygous. (*E*) Body weights of the mice shown in *D* (mean and standard deviation). Two-way repeated measures of ANOVA were performed (weeks 3 and 40 were excluded, as not all animals were analyzed; one wild-type and two heterozygous animals that died were also excluded). *n* = 8 wild type; *n* = 8 heterozygous; *n* = 10 homozygous. Genotype effect: *F*_(2,23)_ = 0.3047. *P* = 0.7403. (*F*) Clasping score of the mice shown in *D*. Two-way repeated measures of ANOVA were performed (weeks 3 and 40 were excluded, as not all animals were analyzed; one wild-type and two heterozygous animals that died were also excluded). *n* = 8 wild type; *n* = 8 heterozygous; *n* = 10 homozygous. Genotype effect: *F*_(2,23)_ = 4.964. *P* = 0.0161. Overall scores are in Supplemental Figure S4G. (*G*) Western blots of three brains of mice heterozygous and homozygous for *Tau-Mecp2[R133C]* and wild-type littermates (males, 7 wk) probed for MeCP2 and γ-tubulin. (*H*) Quantification of blots shown in *G* (mean and standard deviation). The genotypes used were *+/+* (black), *+/Tau-Mecp2[R133C]* (cyan), and *Tau-Mecp2[R133C]/Tau-Mecp2[R133C]* (dark blue). A two-tailed unpaired *t*-test was used for statistical analysis. *P* = 0.0028 for wild type versus heterozygous; *P* = 0.0002 for wild type versus homozygous. (*I*) Genotypes resulting from *Tau-Mecp2[R133C]* heterozygote intercrosses observed at weaning. Legend is as for *H*. A χ^2^ test was used to test for normal Mendelian ratios of genotypes. *P* = 0.6256. (*J*) Survival of a cohort comprising *+/+* (black; *n* = 11), *+/Tau-Mecp2[R133C]* (cyan; *n* = 10), and *Tau-Mecp2[R133C]/Tau-Mecp2[R133C]* (dark blue; *n* = 12). This was analyzed using a Mantel-Cox test. *P* = 0.2943 for wild type versus heterozygous; *P* = 0.1560 for wild type versus homozygous. (*K*) Body weights of the mice shown in *J* (mean and standard deviation). Two-way repeated measures of ANOVA were performed (week 3 was excluded as not all animals were analyzed; one heterozygous and three homozygous animals that died were also excluded). *n* = 11 wild type; *n* = 9 heterozygous; *n* = 9 homozygous. Genotype effect: *F*_(2,26)_ = 5.937. *P* = 0.0075. (*L*) Clasping score of the mice shown in *J*. Two-way repeated measures of ANOVA were performed (week 3 was excluded, as not all animals were analyzed; one heterozygous and three homozygous animals that died were also excluded). *n* = 11 wild type; *n* = 9 heterozygous; *n* = 9 homozygous. Genotype effect: *F*_(2,26)_ = 16.6. *P* < 0.0001.

We next performed the equivalent experiment with mutations that affect DNA binding via the MBD. We first introduced the most common missense Rett syndrome mutation, T158M (which compromises DNA binding but also reduces MeCP2 stability), into the Tau locus (Supplemental Fig. S4A,B; Brown et al. 2016). As anticipated, mice heterozygous for the *Tau-Mecp2[T158M]* allele that also lacked endogenous MeCP2 exhibited Rett syndrome-like phenotypes similar in severity to equivalent mutations at the endogenous *Mecp2* locus. Although mice homozygous for the *Tau-Mecp2[T158M]* allele in an *Mecp2*-null background showed improved survival compared with *Mecp2*-null mice, phenotypic scoring was not improved (Supplemental Fig. S4C–F; Brown et al. 2016). Due to instability of the mutant protein, we found that overexpression from the *Tau-Mecp2[T158M]* locus led to only a modest increase in MeCP2 expression, reaching ∼1.8 times wild type in *Tau-Mecp2[T158M]* homozygotes in the presence of endogenous MeCP2 (Fig. 4A,B). This low level of additional mutant protein did not severely affect survival or weight of mice, but hindlimb clasping was strikingly elevated from ∼10 wk of age, indicative of a significant brain pathology. Other phenotypic aspects detected by weekly scoring were not significantly altered (Fig. 4C–F; Supplemental Fig. S4G).

To determine whether MBD mutants consistently caused overexpression phenotypes, we introduced the R133C mutation into the *Tau* locus. R133C is another common Rett syndrome mutation that also reduces DNA binding and abundance of MeCP2, although less severely than T158M (Supplemental Fig. S5A,B; Brown et al. 2016). As in the case of T158M, mice with the *Tau-Mecp2[R133C]* allele in the absence of the endogenous *Mecp2* gene mimicked the phenotype of an endogenous *Mecp2[R133C]* allele (Supplemental Fig. S5C–F). Similar to the previously published knock-in allele, heterozygous or homozygous *Tau-MeCP2[R133C]* mice in an *Mecp2*-null background showed improved survival compared with *Mecp2*-null animals, but their phenotypic scores were not improved. Mice homozygous for *Tau-Mecp2[R133C]* in the presence of endogenous wild-type MeCP2 expressed wild type plus mutant proteins at 2.6 times wild-type and were recovered at normal frequency at weaning (Fig. 4G–I). Although this level of expression is similar to ∼2.4-fold overexpression of wild-type protein in *Tau-Mecp2* heterozygotes, which have no obvious phenotype, we found that excess R133C protein led to pronounced hindlimb clasping beyond ∼6 wk of age. These animals also displayed subtly reduced body weight, although no significant effect on survival was detected (Fig. 4J–L; Supplemental Fig. S5G). Thus, when expressed in addition to wild-type MeCP2, the toxicity observed previously for T158M commenced earlier with the R133C mutation. We conclude that, in contrast to overexpression of the R306C mutant protein, which is benign even at high levels, relatively modest overexpression of MeCP2 with missense MBD mutations in a brain that additionally has normal levels of wild-type MeCP2 is markedly deleterious.

### The toxicity of MeCP2 overexpression is independent of HDAC3 activity

**Figure 5.**
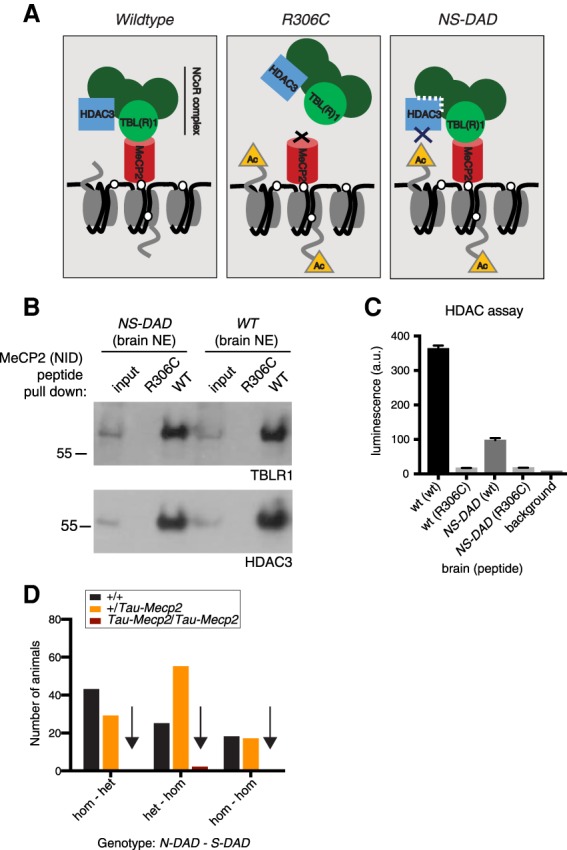
Reducing HDAC3 activity does not rescue lethality of *Tau-Mecp2* overexpression. (*A*) Schematic overview of the experiment. In wild-type brains, MeCP2 (red cylinder) recruits the core NCoR1/2 complexes (green circles) by binding to TBL1X(R1) (bright-green circle). If the NID of MeCP2 is mutated (R306C), MeCP2 cannot bind and recruit the NCoR complexes. In *NS-DAD* mutants, the mutated DADs of both NCoR1 and NCoR2 fail to fully activate HDAC3, allowing removal of histone acetyl groups. (*B*) An MeCP2 peptide containing either the wild-type or a mutated (R306C) NID was used to pull down TBL1XR1 and HDAC3 from brain nuclear extracts from wild-type or NS-DAD animals. Wild-type MeCP2 pulls down NCoR1/2 components from both wild-type and *NS-DAD* brain extracts with similar efficiency, whereas the R306C peptide is unable to pull down the corepressor subunits from either extract. (*C*) Proteins isolated in *B* were assayed for HDAC activity. Shown are mean and standard deviation of three biological replicates as well as background luminescence readings. HDAC activity of proteins pulled down using an MeCP2 wild-type peptide from *NS-DAD* brains is reduced to ∼27% of wild-type brains. The R306C peptide recovered ∼5% HDAC activity irrespective of brain genotype. (*D*) The number of animals surviving to weaning when wild-type, heterozygous or homozygous for *Tau-Mecp2*, and either heterozygous or homozygous for *N-DAD* and *S-DAD* mutations. The failure of animals homozygous for *Tau-Mecp2* to survive was not rescued by reducing HDAC3 activity via DAD mutations either singly or on both NCoR1 and NCoR2. For survival of additional genotypes, see Supplemental Figure S6A. Note that as *Ncor* and *Tau* genes are located on the same chromosome (42 Mb apart), *+/Tau-Mecp2* is observed at a reduced frequency if *N-DAD* is homozygous (43 cM). A χ^2^ test was used to test for normal Mendelian ratio of genotypes. *P* < 0.0001 for homozygous–heterozygous; *P* = 0.0001 for heterozygous–homozygous; *P* = 0.0025 for homozygous–homozygous, indicating a non-Mendelian distribution.

Our finding that inactivation of the NID via an R306C mutation abolishes the toxicity caused by MeCP2 overexpression focuses attention on the function of this domain. Previous evidence supports the hypothesis that its primary role is to bind the TBL1X(R1) subunit and thereby recruit NCoR corepressors (Lyst et al. 2013; Kruusvee et al. 2017). A key shared subunit of NCoR1/2 complexes is HDAC3 (You et al. 2013). HDAC3 is considered to be the component of NCoR1/2 complexes that is responsible for inhibiting transcription via deacetylation of histones. To determine genetically whether the activity of HDAC3 mediates the toxic effects of MeCP2 overexpression, we used mutants in NCoR1 and NCoR2 proteins that reduce HDAC3 catalytic activity (Fig. 5A; You et al. 2013). HDAC3 is catalytically inactive in isolation but becomes active upon interaction with the DADs of NCoR1 and NCoR2 proteins (Guenther et al. 2001; You et al. 2013). The DAD mutations in NCoR1/2 greatly reduce HDAC3 activation, but the reported phenotype of homozygous double mutants (NS-DAD mutants) is mild without overt neurological abnormalities (You et al. 2013). If HDAC3 activity within the NCoR1/2 complexes is the primary downstream effector of MeCP2, these mice should at a minimum exhibit Rett syndrome-like features but in fact are viable with normal survival. As HDAC3-null mutations are embryonically lethal, we considered the possibility that DAD mutations retain some HDAC3 activity. We recovered NCoR1/2 complexes from brain extracts using affinity for a NID peptide and found that HDAC3 was retrieved equally from wild-type and NS-DAD mutant extracts, indicating no detectable effect of the mutations on the binding of HDAC3 to NCoR1 or NCoR2 complexes (Fig. 5B). However, the amount of recovered HDAC activity was drastically reduced in NS-DAD mutant brains (Fig. 5C). We conclude that the DAD mutations significantly reduce HDAC activity associated with NCoR complexes by >70%.

The finding that the DAD mutations show severely reduced HDAC activity made it possible to test whether the toxicity caused by MeCP2 overexpression is due to excessive HDAC3 activity. According to this hypothesis, DAD mutations would be expected to significantly reduce the severity of this phenotype. The experiment requires MeCP2 overexpression in mice that are homozygous for knock-in DAD mutations in both NCoR1 and NCoR2. Despite the reduced ability of the DAD mutant corepressors to activate HDAC3, we observed no discernible improvement in the early lethal phenotype resulting from MeCP2 overexpression (Fig. 5D; Supplemental Fig. S6A). Therefore, in contrast to overexpression of the R306C mutant, which resulted in effectively wild-type mice, the inability of NCoR1/2 to fully activate HDAC3 did not ameliorate the overexpression phenotype. Our results suggest that while toxicity very likely requires excessive TBL1XR1 and hence NCoR1/2 complex recruitment, it does not depend on HDAC3 activity.

## Discussion

A goal of the present work was to determine which domains of the MeCP2 protein underlie the severe phenotype caused by its overexpression. To achieve this, we drove transcription of an MeCP2 cDNA from the endogenous *Tau* locus in mice that also expressed the X-linked wild-type gene. Levels of Tau-MeCP2 closely mimicked those of endogenous MeCP2 in both neurons and glia. In agreement with a previous report (Luikenhuis et al. 2004), mice with only Tau-MeCP2 as a source of MeCP2 protein were viable, fertile, and overtly normal in phenotype despite lacking the native promoter and the unusually long (∼8.5-kb) 3′ untranslated region that is an evolutionarily conserved feature of the endogenous transcript (Singh et al. 2008). When this autosomal transgene was expressed heterozygously in mice with an endogenous *Mecp2* gene, animals were also phenotypically relatively normal despite a 2.4-fold increase in brain MeCP2 (Supplemental Fig. S6B). The results raise the possibility that mice are more tolerant than humans to MeCP2 overexpression. It was reported previously that expression of the human *MECP2* locus in mice with normal levels of the endogenous protein also leads to a relatively mild phenotype, although survival and motor function are compromised to a somewhat greater extent than seen here (Collins et al. 2004). The difference may be due to the altered genetic background or brain region-specific expression in those experiments.

A recent study provided evidence that twofold overexpression of MeCP2 carrying either the R306C mutation, which abolishes NID function, or the R111G mutation, which abolishes binding to methylated DNA, is phenotypically neutral (Heckman et al. 2014). The severe phenotype caused by higher expression of Tau-MeCP2 provides a more stringent test of the toxicity of mutant MeCP2 overexpression. Using this approach, we establish here that ∼3.6-fold overexpression of MeCP2[R306C] leads to a wild-type phenotype, in stark contrast to the lethal phenotype caused by equivalent levels of wild-type MeCP2 (Supplemental Fig. S6B). It follows that toxicity is intimately dependent on excess of this domain, whose only well-characterized function is to bind the WD40 domain of TBL1X and its close relative, TBL1XR1. Indeed, a recent crystal structure of the NID bound to the TBL1XR1 subunit, which is shared by NCoR1/2 complexes, showed that four adjacent amino acids that are individually mutated in Rett syndrome (one of which is R306) all make intimate molecular contacts with the WD40 domain of TBL1XR1 (Kruusvee et al. 2017). Our data suggest that the MeCP2–TBL1X(R1) interaction, which is critical for avoidance of Rett syndrome, is also of pivotal importance as a cause of *MECP2* duplication syndrome. It seems that Rett syndrome is caused by too little recruitment of TBL1X(R1) to DNA, whereas in *MECP2* duplication syndrome, TBL1X(R1) recruitment is excessive.

TBL1X(R1) proteins are best known as components of the NCoR1/2 corepressor complexes, where they form a tetramer that associates with the NCoR scaffolds (Oberoi et al. 2011). As MeCP2 pulls down major subunits of NCoR1/2 complexes by immunoprecipitation (Lyst et al. 2013), we infer that the primary consequence of interaction with TBL1X(R1) is recruitment of these corepressors. While it is conceivable that TBL1X(R1) is involved in additional functional complexes that are relevant to Rett syndrome, none has so far been characterized. We therefore sought to establish what features of NCoR1/2 might account for toxicity. The primary mediator of transcriptional inhibition by NCoR corepressors is thought to be histone deacetylation via HDAC3, which is inactive unless intimately associated with these complexes (Guenther et al. 2001; You et al. 2013). In view of evidence that occupancy of gene bodies by MeCP2 in vivo correlates positively with relative down-regulation of gene expression (Kinde et al. 2016; Lagger et al. 2017), an attractive possibility was that this effect is mediated through histone deacetylation by HDAC3. Based on our evidence that hyperrecruitment of TBL1X(R1) underlies the MeCP2 overexpression phenotype, it follows that reducing HDAC3 activity associated with NCoR1/2 complexes might ameliorate this phenotype. Strikingly, though, we saw no reduction in the severe consequences of Tau-MeCP2 overexpression in NS-DAD mice. Although absence of a genetic rescue must be interpreted with caution, the fact that DAD mutations in both NCoR1 and NCoR2 (You et al. 2013) reduced MeCP2-associated HDAC activity by ∼72% (Fig. 5C) strongly suggests that the MeCP2 overexpression phenotype is independent of HDAC3 activity. It is likely that even a small reduction in severity would have been detected in this experiment, as MeCP2-overexpressing mice are born but fail to survive until weaning. For example, our *Tau-Mecp2* homozygous mice on a wild-type background (total of 3.8-fold overexpression) fail to survive until weaning, whereas *Tau-Mecp2* homozygous mice on a *Mecp2*-null background (2.8-fold overexpression) were recovered at Mendelian ratios (Fig. 2D; Supplemental Fig. S2J). The finding that this ∼26% reduction in MeCP2 dosage is sufficient for a striking improvement in phenotypic outcome suggests that the phenotype is sensitive enough to detect subtle decreases in the level of MeCP2 functionality. Taken together, the results lead us to conclude that HDAC3 activity does not play a major part in MeCP2 toxicity. It follows that inhibitors of HDAC3 are unlikely to be therapeutically useful for treatment of *MECP2* duplication syndrome. An alternative strategy might be to inhibit the interaction of MeCP2 with NCoR1/2 complexes themselves using small molecules.

Assuming that the interaction between MeCP2 and TBL1X(R1) predominantly recruits NCoR1/2, we may ask what other features of these corepressors might be responsible for the adverse phenotype. Interestingly, the HDAC3 protein itself has been reported to affect transcription in a deacetylation-independent manner (Sun et al. 2013), which probably would not be affected by the DAD mutations. Also, it is possible that other enzymes, notably class IIa HDACs, associate with NCoR either directly or indirectly and cause deacetylation (Fischle et al. 2001; Fischle et al. 2002; Hudson et al. 2015). A further possibility is that the large NCoR1/2 complexes have other functions that mediate the deleterious consequences of overrecruitment. These could include recruiting activities such as the ubiquitin protease USP44 (Lan et al. 2016) or the ubiquitin-conjugating enzyme UbcH5 (Perissi et al. 2004). Distinguishing these possibilities is a priority for future work.

Unlike MeCP2[R306C] overexpression, which was phenotypically neutral, mild overexpression of Rett syndrome-causing MBD mutants on top of wild-type MeCP2 was detrimental. Both T158M and R133C mutations are defective in DNA binding but also somewhat unstable. To our knowledge, all MBD mutants of MeCP2 reported so far are unstable to varying degrees. Because of this, overexpression did not yield the high levels seen with wild-type or R306C mutant proteins. Despite very mild overexpression, we observed a strong hindlimb-clasping phenotype in each case, indicative of pathologies affecting the brain and/or spinal cord (Lalonde and Strazielle 2011). The toxic effect was stronger in the case of R133C, which retains more DNA-binding affinity, is more stable than T158M, and gave ∼2.6 times wild-type levels of total MeCP2 in brain. In contrast, animals expressing equivalent levels of wild-type protein (2.4 times wild type) were phenotypically normal apart from reduced weight. These findings raise the possibility that overexpression of a protein that cannot effectively bind DNA causes dominant-negative effects. In this connection, it may be relevant that both of these proteins contain an intact NID, which may still be available to interact with NCoR1/2. A previous study using a different MBD mutation, R111G, expressed on top of wild-type MeCP2 led to a different conclusion, as no adverse phenotype was observed (Heckman et al. 2014). The disparity is unexplained but may be due to differences in genetic background, the alternative mutants that were analyzed, or the nonoverlapping assay paradigms used in the two cases. Whether the toxicity of MBD mutated MeCP2 requires HDAC3 activity has yet to be formally tested, although we consider this unlikely given that wild-type MeCP2 appears not to function via this pathway.

A separate study suggested that overexpression of T158M in an *Mecp2*-null background ameliorated phenotypic severity and may therefore offer a potential therapeutic strategy for Rett syndrome (Lamonica et al. 2017). Due to the instability of the mutant protein, the risk of toxicity caused by MeCP2 overexpression would arguably be reduced. Our results cast doubt on the validity of this approach, as mice expressing a combination of wild-type and mutant protein were adversely affected compared with mice expressing the same level of only wild-type MeCP2. As the Rett syndrome brain is mosaic for functionally wild-type and mutant cells, there is a risk that delivery of excess T158M or R133C mutant proteins would cause toxic side effects. Another concern raised by these results is that future gene therapy in which wild-type MeCP2 would be introduced into neurons expressing an MBD mutant form (e.g., T158M) would mimic the protein combination that gives a hindlimb-clasping phenotype in our study. Further work with model systems is needed to determine whether gene therapy for individuals with MBD mutations might carry a risk and whether this would be outweighed by clinical benefit.

## Materials and methods

### Generation of knockout embryonic stem cells and mice

The targeting vector containing a homology region around *Tau* exon 2 and an in-frame insertion of *Mecp2* cDNA (e2 isoform) after the first 31 amino acids of the *Tau*-coding sequence (a gift from Dr Rudolf Jaenisch) (Luikenhuis et al. 2004) was modified in the following way: To introduce the 3′ loxP site, a neomycin resistance cassette driven by a hybrid PGK-EM7 promoter with an upstream loxP site from PL452 (Liu et al. 2003) replaced the previous neomycin resistance cassette by recombineering. The 5′ *loxP* site was inserted by standard cloning of a *loxP*-containing oligo into the BplI site upstream of *Tau* exon 2. The 5′ homology arm was extended by integrating a KpnI fragment (mm9: chromosome 11: 104,142,690–104,143,022) obtained by PCR from E14 Tg2a embryonic stem cell DNA. To integrate point mutations within *Mecp2* (T158M, R133C, and R306C), the QuikChange II XL site-directed mutagenesis kit (Stratagene, 200521) was used (primers: R306C-S [TCCCCATCAAGAAGTGCAAGACCCGGGAG], R306C-AS [CTCCCGGGTCTTGCACTTCTTGATGGGGA], R133C-S [CCAGGGAAAAGCTTTTTGCTCTAAAGTAGAATTG], R133C-AS [CAATTCTACTTTAGAGCAAAAAGCTTTTCCCTGG], T158M-S [GGACCCTAATGATTTTGACTTCATGGTAACTGGGAGAG], and T158M-AS [CTCTCCCAGTTACCATGAAGTCAAAATCATTAGGGTCC]). The targeting vector was linearized using SacII before electroporation. Electroporation of the targeting vectors and neomycin selection were performed using standard conditions in E14 Tg2a (129P2/OlaHsd) embryonic stem cells. Embryonic stem cells were grown in Glasgow MEM (Gibco) supplemented with 10% FBS (batch tested) (Gibco), 1% nonessential amino acids (Gibco), 1% sodium pyruvate (Gibco), 0.1% β-mercaptoethanol (Gibco), and 1000 U/mL LIF (ESGRO). Positive embryonic stem cell clones were identified by Southern blot screening and PCR fragment sequencing confirmation. They were injected into C57BL/6JOla blastocysts and transferred into pseudo-pregnant recipient mice. Transgenic offspring were backcrossed onto C57BL/6JOla or crossed with *Mecp2*-null animals on a C57BL/6JOla × CBA F1 background for further experiments. Tau knockout mice were generated by mating Tau-Mecp2 mice with CMV-CRE mice (JAX, 006054) to delete the sequence in between the two *loxP* sites.

All mice used in this study were bred and maintained at the University of Edinburgh animal facility under standard conditions, and procedures were carried out by staff licensed by the UK Home Office and in accordance with the Animal and Scientific Procedures Act 1986. Transgenic mice were caged with their wild-type littermates.

### Biochemical analysis

Brains (usually at 6–8 wk of age) were harvested by snap-freezing in liquid nitrogen. DNA isolation was performed by overnight incubation in 1× TEN (50 mM Tris at pH 9.0, 20 mM EDTA at pH 8.0, 40 mM NaCl, 1% SDS, 0.5 mg/mL proteinase K) at 55°C followed by high-salt extraction and precipitation using isopropanol. For Southern blots, 10- to 20-µg aliquots of genomic DNA were digested overnight with 2 U/µg suitable restriction enzyme and separated by gel electrophoresis on 0.8% agarose gels and 1× TBE at 5.3 V/cm. The gel was denatured twice for 30 min in denaturing solution (0.5 M NaOH, 1.5 M NaCl) and blotted for at least 18 h onto Hybond-XL nylon membranes (GE Healthcare) in denaturing solution. After neutralization of the membrane in 20 mM Na_2_HPO_4_, the membrane was prehybridized in Church buffer (0.25M Na_2_HPO_4_, 7% SDS, 1 mM EDTA) for 30 min at 65°C followed by hybridization with radioactive probes in Church buffer for at least 18 h at 65°C. Probes (Tau-P3: chromosome 11: 104,148,301–104,149,009; Tau-P4: chromosome 11: 104,142,739–104,143,549) were labeled using the Prime-a-Gene labeling system (Promega, U1100) with α32-dCTP. Blots were washed twice for 30 min in Church wash (1% SDS, 20 mM Na_2_HPO_4_) and exposed overnight to phosphorimager plates. Plates were scanned using a Typhoon FLA 9500 (GE Healthcare).

Brain nuclear extracts were prepared as described previously (Lyst et al. 2013). HDAC3-containing complexes were isolated using a biotin-tagged MeCP2-derived peptide (residues 285–313) immobilized on streptavidin sepharose (GE Healthcare) as described previously (Lyst et al. 2013). Bound proteins were analyzed by SDS-PAGE followed by Western blotting or by using the HDAC-Glo(TM) I/II assay and screening system (Promega) according to the manufacturer's instructions.

Flow cytometry analysis was performed as described previously (Tillotson et al. 2017) using a NeuN antibody conjugated to AF488 (Millipore, MAB377X) and an MeCP2 antibody (Sigma, M6818) conjugated to AF647 (APEX antibody labeling kit, Invitrogen, A10475). For the immunofluorescent stainings, mouse brains were hemisected and rapidly frozen by immersion in isopentane chilled on dry ice. A Leica CM1900 cryostat was used to cut 10-µm parasagittal sections, which were mounted on SuperFrost Plus slides (VWR) and stored at −80°C prior to staining. Slides for staining were allowed to dry for 10 min at room temperature, fixed in a 1:1 methanol:actetone mixture at −20°C for 20 min, and then dried for 5 min at room temperature. After two 10-min washes in PBS, sections were blocked for 1 h in 1.5% normal goat serum (Sigma) in PBS (blocking buffer) at room temperature in a humidified chamber. The following primary antibodies were incubated overnight at 4°C: anti-MeCP2 rabbit monoclonal D4F3 (Cell Signalling Technology) and anti-NeuN Cy3-labeled mouse monoclonal A60 (EMD Millipore Corp.), both diluted 1:200 in blocking buffer. Sections were washed as described previously and then incubated with a biotinylated anti-rabbit secondary antibody (Vector Laboratories) diluted 1:200 in blocking buffer for 1.5 h at room temperature. After washing again, sections were incubated with avidin-FITC (Vector Laboratories) diluted 1:200 in 10 mM HEPES and 150 mM NaCl for 20 min at room temperature. Sections were then washed three times for 10 min in PBS, with the first wash including 1 µg/mL DAPI. They were then mounted in ProLong Gold (Life Technologies), left to cure in the dark overnight at room temperature, and then stored at 4°C. Stained sections were imaged on a Leica SP5 confocal microscope using a 63× objective, keeping all settings the same between samples (apart from R133C and T158M high-exposure images, where the gain was increased [by the same amount] for the FITC channel to visualize the low levels of MeCP2).

### Phenotypic characterization of mice

Male mice on a mixed background from the age of 3–4 wk until 36–52 wk were subjected to a weekly scoring and weighing regime as described in Brown et al. (2016). Animals that are described to having “died” in most instances were culled having reached one of the end points as set out in the Home Office Project Licence. For behavioral analysis using the elevated plus maze and the accelerating rotarod, mice were backcrossed onto C57BL/6JOla for seven generations and analyzed blind to genotype. Males aged 19–20 wk were used for these experiments as described in Brown et al. (2016).

### Statistical analysis

Statistical tests appropriate to the nature of the data were carried out using Graphpad Prism. The death of animals that needed to be culled during the course of the study due to phenotype-unrelated causes (injuries caused by fighting) was censored in the survival analysis. This is indicated by small vertical ticks in the survival curves. To allow analysis by two-way repeated measures of ANOVA on weight and phenotypic scoring data sets where a small number of animals had died during the study, these animals were removed from the analysis. Similarly, weeks when it was not possible to score all of the animals were also removed from the analysis.

## Supplementary Material

Supplemental Material

## References

[GAD320325KOEC1] Amir RE, Van den Veyver IB, Wan M, Tran CQ, Francke U, Zoghbi HY. 1999 Rett syndrome is caused by mutations in X-linked MECP2, encoding methyl-CpG-binding protein 2. Nat Genet 23: 185–188. 10.1038/13810 10508514

[GAD320325KOEC2] Brown K, Selfridge J, Lagger S, Connelly J, De Sousa D, Kerr A, Webb S, Guy J, Merusi C, Koerner MV, 2016 The molecular basis of variable phenotypic severity among common missense mutations causing Rett syndrome. Hum Mol Genet 25: 558–570. 10.1093/hmg/ddv496 26647311 PMC4731022

[GAD320325KOEC3] Collins AL, Levenson JM, Vilaythong AP, Richman R, Armstrong DL, Noebels JL, David Sweatt J, Zoghbi HY. 2004 Mild overexpression of MeCP2 causes a progressive neurological disorder in mice. Hum Mol Genet 13: 2679–2689. 10.1093/hmg/ddh282 15351775

[GAD320325KOEC4] Cuddapah VA, Pillai RB, Shekar KV, Lane JB, Motil KJ, Skinner SA, Tarquinio DC, Glaze DG, McGwin G, Kaufmann WE, 2014 Methyl-CpG-binding protein 2 (MECP2) mutation type is associated with disease severity in Rett syndrome. J Med Genet 51: 152–158. 10.1136/jmedgenet-2013-102113 24399845 PMC4403764

[GAD320325KOEC5] Dawson HN, Ferreira A, Eyster MV, Ghoshal N, Binder LI, Vitek MP. 2001 Inhibition of neuronal maturation in primary hippocampal neurons from τ deficient mice. J Cell Sci 114: 1179–1187. 10.1242/jcs.114.6.1179 11228161

[GAD320325KOEC6] Fischle W, Dequiedt F, Fillion M, Hendzel MJ, Voelter W, Verdin E. 2001 Human HDAC7 histone deacetylase activity is associated with HDAC3 *in vivo*. J Biol Chem 276: 35826–35835. 10.1074/jbc.M104935200 11466315

[GAD320325KOEC7] Fischle W, Dequiedt F, Hendzel MJ, Guenther MG, Lazar MA, Voelter W, Verdin E. 2002 Enzymatic activity associated with class II HDACs is dependent on a multiprotein complex containing HDAC3 and SMRT/N-CoR. Mol Cell 9: 45–57. 10.1016/S1097-2765(01)00429-4 11804585

[GAD320325KOEC8] Guenther MG, Barak O, Lazar MA. 2001 The SMRT and N-CoR corepressors are activating cofactors for histone deacetylase 3. Mol Cell Biol 21: 6091–6101. 10.1128/MCB.21.18.6091-6101.2001 11509652 PMC87326

[GAD320325KOEC9] Harada A, Oguchi K, Okabe S, Kuno J, Terada S, Ohshima T, Sato-Yoshitake R, Takei Y, Noda T, Hirokawa N. 1994 Altered microtubule organization in small-calibre axons of mice lacking tau protein. Nature 369: 488–491. 10.1038/369488a0 8202139

[GAD320325KOEC10] Heckman LD, Chahrour MH, Zoghbi HY. 2014 Rett-causing mutations reveal two domains critical for MeCP2 function and for toxicity in MECP2 duplication syndrome mice. Elife 3 10.7554/eLife.02676 PMC4102243 24970834

[GAD320325KOEC11] Hudson GM, Watson PJ, Fairall L, Jamieson AG, Schwabe JW. 2015 Insights into the recruitment of class IIa histone deacetylases (HDACs) to the SMRT/NCoR transcriptional repression complex. J Biol Chem 290: 18237–18244. 10.1074/jbc.M115.661058 26055705 PMC4505066

[GAD320325KOEC12] Kinde B, Wu DY, Greenberg ME, Gabel HW. 2016 DNA methylation in the gene body influences MeCP2-mediated gene repression. Proc Natl Acad Sci 113: 15114–15119. 10.1073/pnas.1618737114 27965390 PMC5206576

[GAD320325KOEC13] Kruusvee V, Lyst MJ, Taylor C, Tarnauskaite Z, Bird AP, Cook AG. 2017 Structure of the MeCP2-TBLR1 complex reveals a molecular basis for Rett syndrome and related disorders. Proc Natl Acad Sci 114: E3243–E3250. 10.1073/pnas.1700731114 28348241 PMC5402415

[GAD320325KOEC14] Lagger S, Connelly JC, Schweikert G, Webb S, Selfridge J, Ramsahoye BH, Yu M, He C, Sanguinetti G, Sowers LC, 2017 MeCP2 recognizes cytosine methylated tri-nucleotide and di-nucleotide sequences to tune transcription in the mammalian brain. PLoS Genet 13: e1006793 10.1371/journal.pgen.1006793 28498846 PMC5446194

[GAD320325KOEC15] Lalonde R, Strazielle C. 2011 Brain regions and genes affecting limb-clasping responses. Brain Res Rev 67: 252–259. 10.1016/j.brainresrev.2011.02.005 21356243

[GAD320325KOEC16] Lamonica JM, Kwon DY, Goffin D, Fenik P, Johnson BS, Cui Y, Guo H, Veasey S, Zhou Z. 2017 Elevating expression of MeCP2 T158M rescues DNA binding and Rett syndrome-like phenotypes. J Clin Invest 127: 1889–1904. 10.1172/JCI90967 28394263 PMC5409785

[GAD320325KOEC17] Lan X, Atanassov BS, Li W, Zhang Y, Florens L, Mohan RD, Galardy PJ, Washburn MP, Workman JL, Dent SYR. 2016 USP44 Is an integral component of N-CoR that contributes to gene repression by deubiquitinating histone H2B. Cell Rep 17: 2382–2393. 10.1016/j.celrep.2016.10.076 27880911 PMC5131803

[GAD320325KOEC18] Lewis JD, Meehan RR, Henzel WJ, Maurer-Fogy I, Jeppesen P, Klein F, Bird A. 1992 Purification, sequence, and cellular localization of a novel chromosomal protein that binds to methylated DNA. Cell 69: 905–914. 10.1016/0092-8674(92)90610-O 1606614

[GAD320325KOEC19] Liu P, Jenkins NA, Copeland NG. 2003 A highly efficient recombineering-based method for generating conditional knockout mutations. Genome Res 13: 476–484. 10.1101/gr.749203 12618378 PMC430283

[GAD320325KOEC20] LoPresti P, Szuchet S, Papasozomenos SC, Zinkowski RP, Binder LI. 1995 Functional implications for the microtubule-associated protein tau: localization in oligodendrocytes. Proc Natl Acad Sci 92: 10369–10373. 10.1073/pnas.92.22.10369 7479786 PMC40798

[GAD320325KOEC21] Luikenhuis S, Giacometti E, Beard CF, Jaenisch R. 2004 Expression of MeCP2 in postmitotic neurons rescues Rett syndrome in mice. Proc Natl Acad Sci 101: 6033–6038. 10.1073/pnas.0401626101 15069197 PMC395918

[GAD320325KOEC22] Lyst MJ, Bird A. 2015 Rett syndrome: a complex disorder with simple roots. Nat Rev Genet 16: 261–275. 10.1038/nrg3897 25732612

[GAD320325KOEC23] Lyst MJ, Ekiert R, Ebert DH, Merusi C, Nowak J, Selfridge J, Guy J, Kastan NR, Robinson ND, de Lima Alves F, 2013 Rett syndrome mutations abolish the interaction of MeCP2 with the NCoR/SMRT co-repressor. Nat Neurosci 16: 898–902. 10.1038/nn.3434 23770565 PMC3786392

[GAD320325KOEC24] Nan X, Meehan RR, Bird A. 1993 Dissection of the methyl-CpG binding domain from the chromosomal protein MeCP2. Nucleic Acids Res 21: 4886–4892. 10.1093/nar/21.21.4886 8177735 PMC311401

[GAD320325KOEC25] Oberoi J, Fairall L, Watson PJ, Yang JC, Czimmerer Z, Kampmann T, Goult BT, Greenwood JA, Gooch JT, Kallenberger BC, 2011 Structural basis for the assembly of the SMRT/NCoR core transcriptional repression machinery. Nat Struct Mol Biol 18: 177–184. 10.1038/nsmb.1983 21240272 PMC3232451

[GAD320325KOEC26] Perissi V, Aggarwal A, Glass CK, Rose DW, Rosenfeld MG. 2004 A corepressor/coactivator exchange complex required for transcriptional activation by nuclear receptors and other regulated transcription factors. Cell 116: 511–526. 10.1016/S0092-8674(04)00133-3 14980219

[GAD320325KOEC27] Singh J, Saxena A, Christodoulou J, Ravine D. 2008 MECP2 genomic structure and function: insights from ENCODE. Nucleic Acids Res 36: 6035–6047. 10.1093/nar/gkn591 18820302 PMC2577328

[GAD320325KOEC28] Sun Z, Feng D, Fang B, Mullican SE, You SH, Lim HW, Everett LJ, Nabel CS, Li Y, Selvakumaran V, 2013 Deacetylase-independent function of HDAC3 in transcription and metabolism requires nuclear receptor corepressor. Mol Cell 52: 769–782. 10.1016/j.molcel.2013.10.022 24268577 PMC3877208

[GAD320325KOEC29] Tillotson R, Selfridge J, Koerner MV, Gadalla KKE, Guy J, De Sousa D, Hector RD, Cobb SR, Bird A. 2017 Radically truncated MeCP2 rescues Rett syndrome-like neurological defects. Nature 550: 398–401. 10.1038/nature24058 29019980 PMC5884422

[GAD320325KOEC30] Van Esch H, Bauters M, Ignatius J, Jansen M, Raynaud M, Hollanders K, Lugtenberg D, Bienvenu T, Jensen LR, Gecz J, 2005 Duplication of the MECP2 region is a frequent cause of severe mental retardation and progressive neurological symptoms in males. Am J Hum Genet 77: 442–453. 10.1086/444549 16080119 PMC1226209

[GAD320325KOEC31] You SH, Lim HW, Sun Z, Broache M, Won KJ, Lazar MA. 2013 Nuclear receptor co-repressors are required for the histone-deacetylase activity of HDAC3 in vivo. Nat Struct Mol Biol 20: 182–187. 10.1038/nsmb.2476 23292142 PMC3565028

