# Supplementary material for: Toxicity of overexpressed MeCP2 is independent of HDAC3 activity
Source: Genes Dev. 2018 Dec 1;32(23-24):1514–24. doi: 10.1101/gad.320325.118 (PMC6295171; doi:10.1101/gad.320325.118)
Supplement: Supplemental Material [file supp_32_23-24_1514__index.html]

Toxicity of overexpressed MeCP2 is independent of HDAC3 activity — Supplemental Material 

# Toxicity of overexpressed MeCP2 is independent of HDAC3 activity

## Supplemental Material

- SupplementalFigures.docx
