# Supplementary material for: Toxicity of overexpressed MeCP2 is independent of HDAC3 activity
Source: Genes Dev. 2018 Dec 1;32(23-24):1514–24. doi: 10.1101/gad.320325.118 (PMC6295171; doi:10.1101/gad.320325.118)
Supplement: Supplemental Material [file supp_gad.320325.118_SupplementalFigures.docx]

**Supplemental Figures, Legends and References**

**Toxicity of over-expressed MeCP2 is independent of HDAC3 activity**

Martha V. Koerner, Laura FitzPatrick, Jim Selfridge, Jacky Guy, Dina De Sousa, Rebekah Tillotson, Alastair Kerr, Zheng Sun, Mitchell A. Lazar, Matthew J. Lyst and Adrian Bird

*
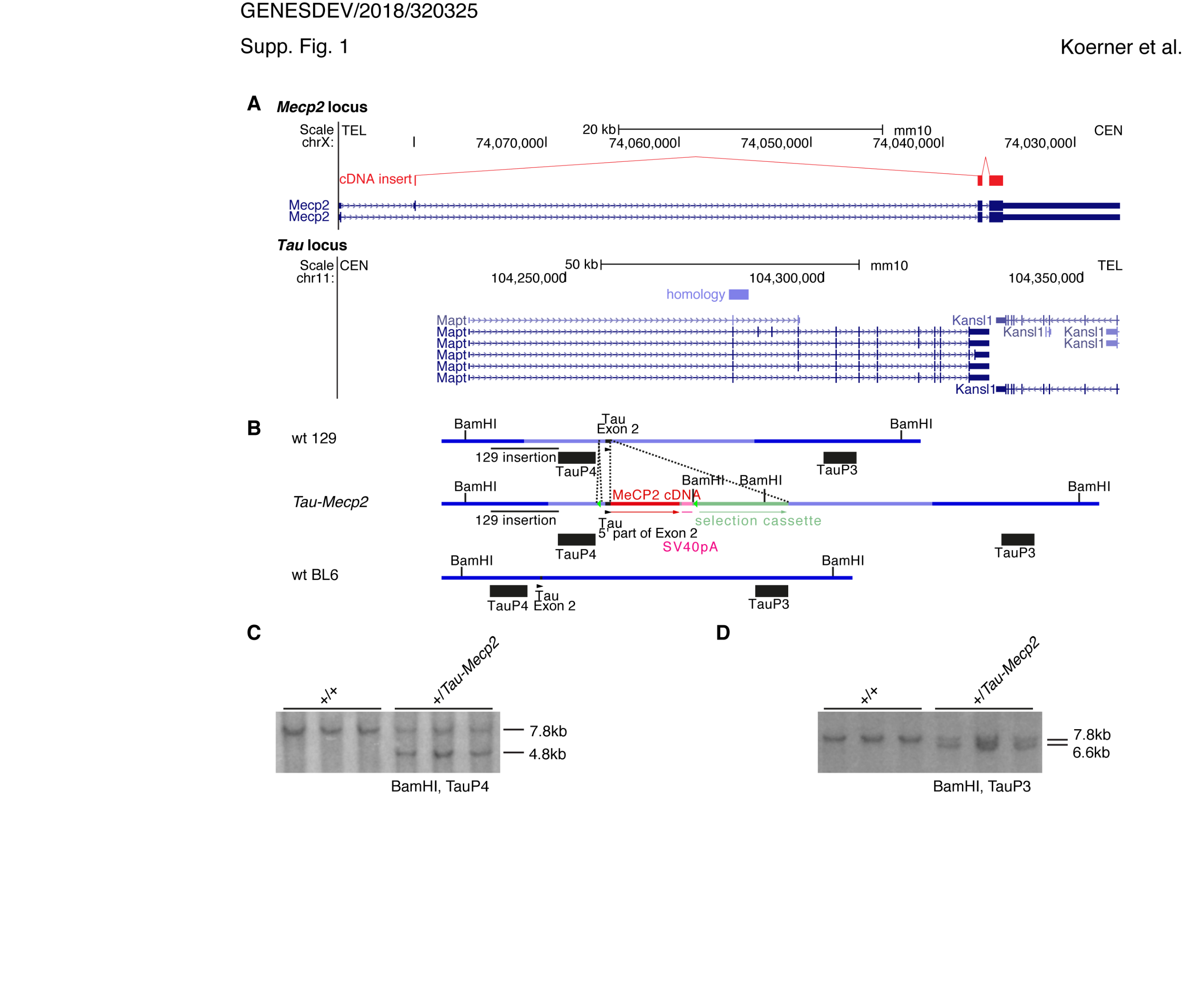
*

*Supp. Fig. 1: Generating Tau-MeCP2 mice.*

1. *Top:* Screenshot from the UCSC genome browser showing the mouse *Mecp2* genomic region. Note that the centromere lies to the right and the telomere end is left. In blue, the 2 MeCP2 isoforms are depicted. Bars denote exons, lines with arrows introns. In red the fragments used for the MeCP2 cDNA insertion into the *Tau* (*Mapt*) locus are shown.

*Bottom:* Screenshot from the UCSC genome browser showing the *Tau* (*Mapt*) genomic region. Different isoforms of *Mapt* and neighbouring gene *Kansl* are depicted. The blue horizontal bar shows the homology region used in the targeting vector.

1. Targeting overview: wt 129/Ola (129) shows the wildtype *Tau* locus as present in the ES cells used for targeting. Pale blue bars denote homology regions, dark blue bars surrounding genomic DNA. Genomic DNA of mouse strain 129 contains a 1.4kb insertion which is absent in C57BL/6 (BL6) genomic DNA. Enzymes (BamHI) and probes (TauP4, TauP3) used for Southern blot analysis are shown.
2. Southern blot of tail genomic DNA for *Tau-Mecp2* mice and wildtype littermates digested with BamHI restriction enzyme and probed with TauP4.
3. Southern blot on BamHI-digested tail DNA for *Tau-Mecp2* mice and wildtype littermates using probe TauP3.

*
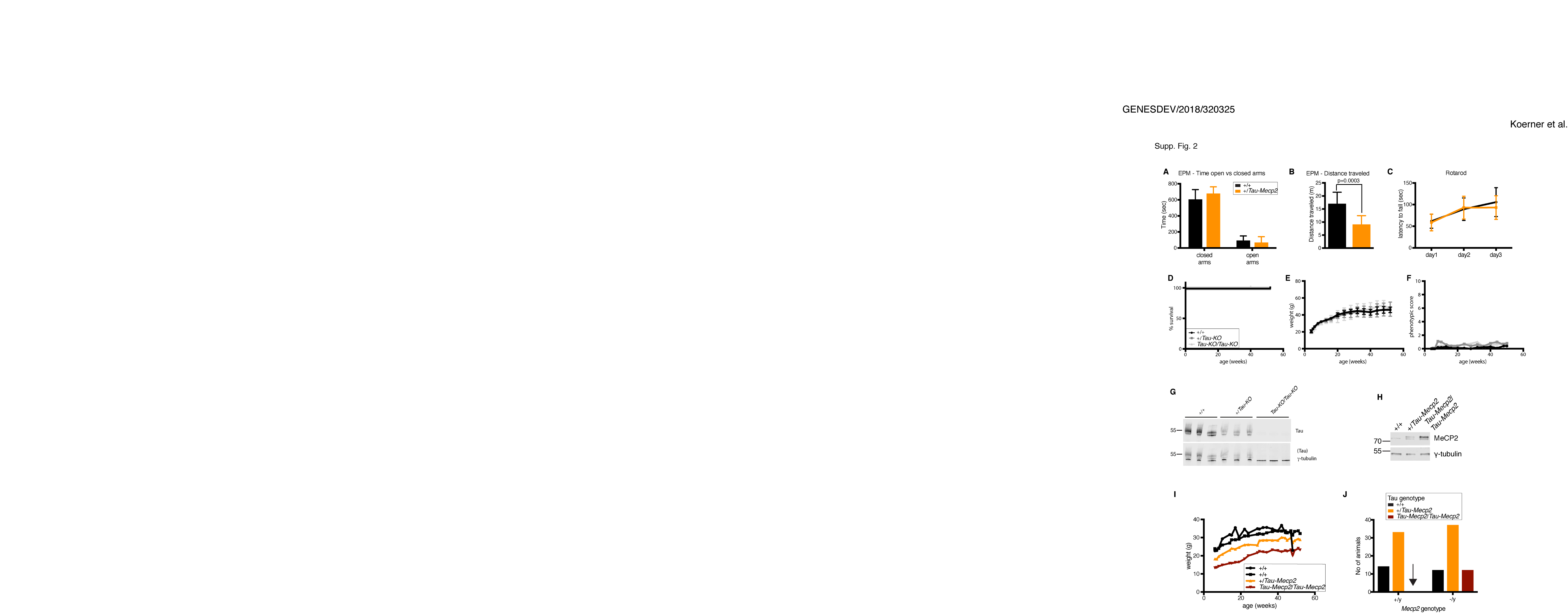
*

*Supp. Fig. 2: 2.4-fold overexpression of MeCP2 is well tolerated, but 3.8-fold is lethal.*

1. +/+ (n=10, black) and +/*Tau-Mecp2* (n=10, orange) animals at 19-20 weeks of age were subject to behavioural analysis using the Elevated Plus Maze (EPM). Time in open *vs* time in closed arms was compared (mean and standard deviation) using an unpaired two-tailed t-test: Time in closed arms: p=0.1472 (ns). Time in open arms: p=0.4436 (ns).
2. As in A) but showing total distance travelled (less in +/*Tau-Mecp2* mice). Unpaired two-tailed t-test: p=0.0003 (****).
3. Motor performance of the animals in A) and B) was analysed using a rotarod. After a learning day the animals were analysed over 3 consecutive days, 4 trials per day. Shown are mean (mean of 4 trials per animal, then the mean of all animals) and standard deviation (of the mean of all animals). Two-way repeated measures ANOVA: F(1,18)=0.1779, p=0.6782 (ns).
4. Survival of a scoring cohort of *Tau*-KO animals. These mice were generated by crossing Tau-MeCP2 animals with a CMV-CRE deleter strain, resulting in the excision of *Tau* exon 2 (containing the ATG) as well as the knocked-in MeCP2 cDNA. Genotypes were: +/+ (n=5, black); +/*Tau*-KO (n=5, dark grey); *Tau*-KO/*Tau*-KO (n=4, pale grey).
5. Body weight of the mice shown in D) (mean and standard deviation). Two-way repeated measures ANOVA (weeks 4-36): Genotype effect F(2,11)=0.1039, P=0.9022 (ns).
6. Phenotypic scores of the mice shown in D). Two-way repeated measures ANOVA (weeks 4-36): Genotype effect F(2,11)=4.972, P=0.0290 (*).
7. Western blots for Tau and γ-tubulin of *Tau*-KO/*Tau*-KO and their heterozygous and wildtype littermates. The blot was first probed with Tau, then stripped and re-probed with γ-tubulin. The still partially visible signal for Tau is indicated by (tau).
8. Western blots for MeCP2 and γ-tubulin of the single surviving *Tau-Mecp2*/*Tau-Mecp2* homozygote and her single heterozygous and wildtype littermate at 52 weeks of age.
9. Weights of mice from H) plus a second wildtype littermate which had to be culled at 48 weeks of age due to severe sudden weight loss.
10. Genotype distribution of +/+ (black), +/*Tau-Mecp2* (orange) and *Tau-Mecp2*/*Tau-Mecp2* animals (red) in animals wildtype for *Mecp2* (+/y) or *Mecp2*-null (-/y). Tau-MeCP2 homozygous mice are recovered at normal Mendelian ratios if the endogenous *Mecp2* allele is mutated. A χ^2^ test was used to test for normal Mendelian ratio of genotypes: *Mecp2 +/y* background: χ^2^=13.57 (2df), p=0.0011 (***). *Mecp2 -/y* background: χ^2^=1.196 (2df), p=0.5499 (ns).

*
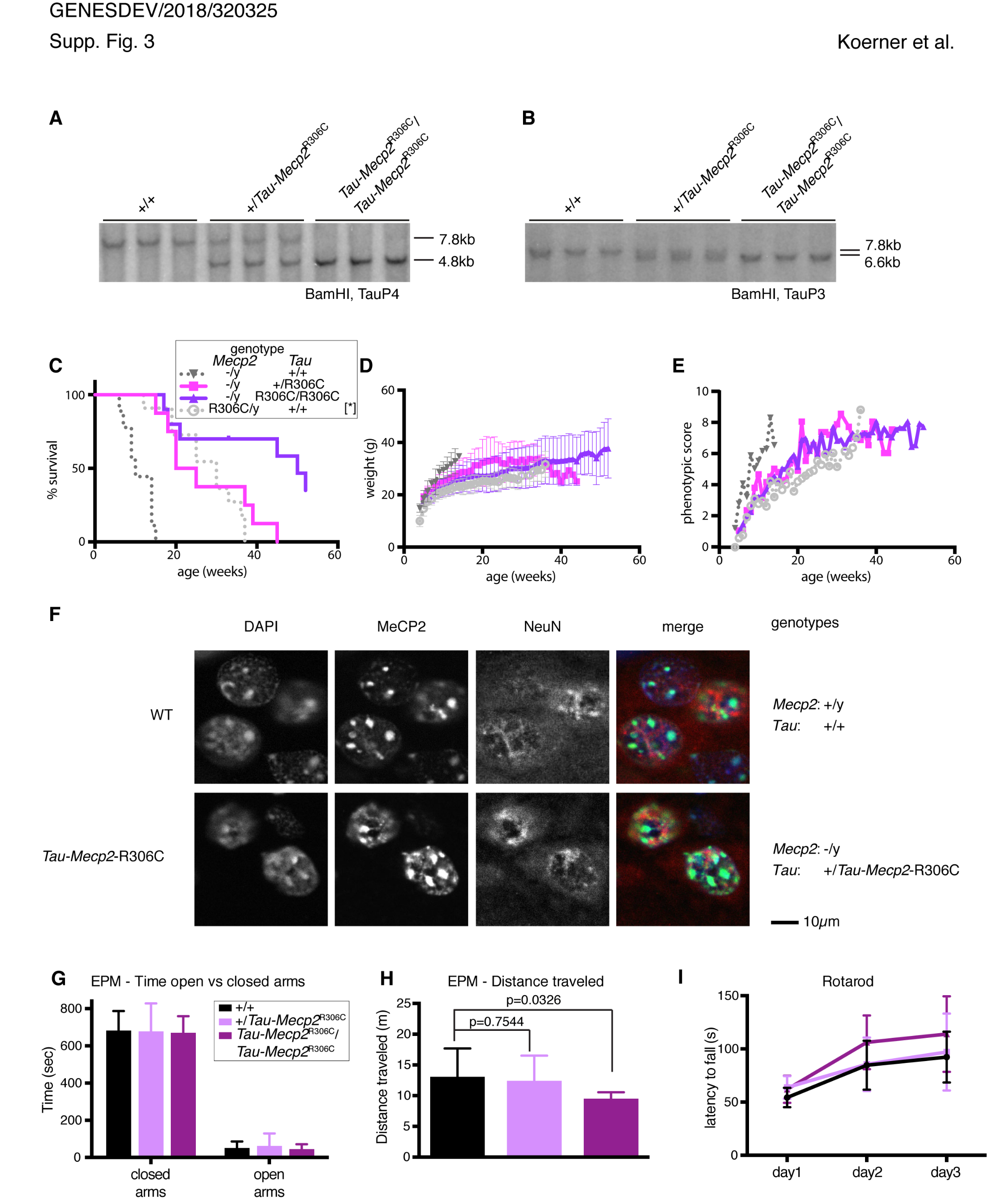
*

*Supp. Fig. 3: A mutation in the MeCP2 NID renders the lethal MeCP2 overdose viable.*

1. Southern blot of BamHI-digested tail DNA for *Tau-Mecp2*[R306C] mice and wildtype littermates using probe TauP4. For locus schematic see Supp. Fig. 1B.
2. Southern blot of BamHI-digested tail DNA for *Tau-Mecp2*[R306C] mice and wildtype littermates using probe TauP3.
3. Survival of cohort comprising *Mecp2* -/y, +/*Tau-Mecp2*[R306C] (n=8, pink); *Mecp2* -/y, *Tau-Mecp2*[R306C]/*Tau-Mecp2*[R306C] (n=10, purple). As comparators, *Mecp2* -/y, +/+ (n=9, dark grey) from an independent cohort and *Mecp2*[R306C] /y, *Tau* +/+ (n=11, light grey) are shown (Brown et al. 2016). This was analysed using a Mantel-Cox test: *Mecp2 -/y, +/Tau-MeCP2[R306C]* vs *Mecp2 -/y, Tau-MeCP2[R306C]/Tau-MeCP2[R306C]*: p=0.0144 (*).
4. Body weight of the mice shown in C) (mean and standard deviation). Due to the high number of animals dying during the study, a statistical analysis was not possible.
5. Phenotypic score of the mice shown in C). Due to the high number of animals dying during the study, a statistical analysis was not possible.
6. Immunofluorescence of a hippocampus CA3 brain section from male mice, 7 weeks of age. Genotypes: wildtype (same image as in Fig. 1E) for comparison, and *Mecp2* -/y, *Tau* +/ *Tau-Mecp2*[R306C]. Sections were stained for DAPI, MeCP2 and NeuN.
7. Behavioural analysis using the Elevated Plus Maze (EPM) of +/+ (n=9, black), +/ *Tau-Mecp2*[R306C] (n=10, light purple) and *Tau-Mecp2*[R306C]/ *Tau-Mecp2*[R306C] (n=10, dark purple) animals at 19-20 weeks of age. Time in open vs closed arms was compared (mean and standard deviation) using an unpaired two-tailed t-tests comparing to WT: Time in closed arms: het p=0.9484 (ns), hom p=0.8024 (ns). Time in open arms: het p=0.6749 (ns), hom p=0.7010 (ns).
8. As in G) but measuring total distance travelled was analysed using an unpaired two-tailed t-test: WT vs het: p=0.7544 (ns) WT vs hom: p=0.0326 (*).
9. The same animals as in G) and H) analysed using a rotarod. After a learning day the animals were analysed over 3 consecutive days, 4 trials per day. Shown are mean (mean of 4 trials per animal, then the mean of all animals) and standard deviation of the mean for all animals, analysed using a two-way repeated measures ANOVA: F(2,26)=1.738, P=0.1957 (ns).

*
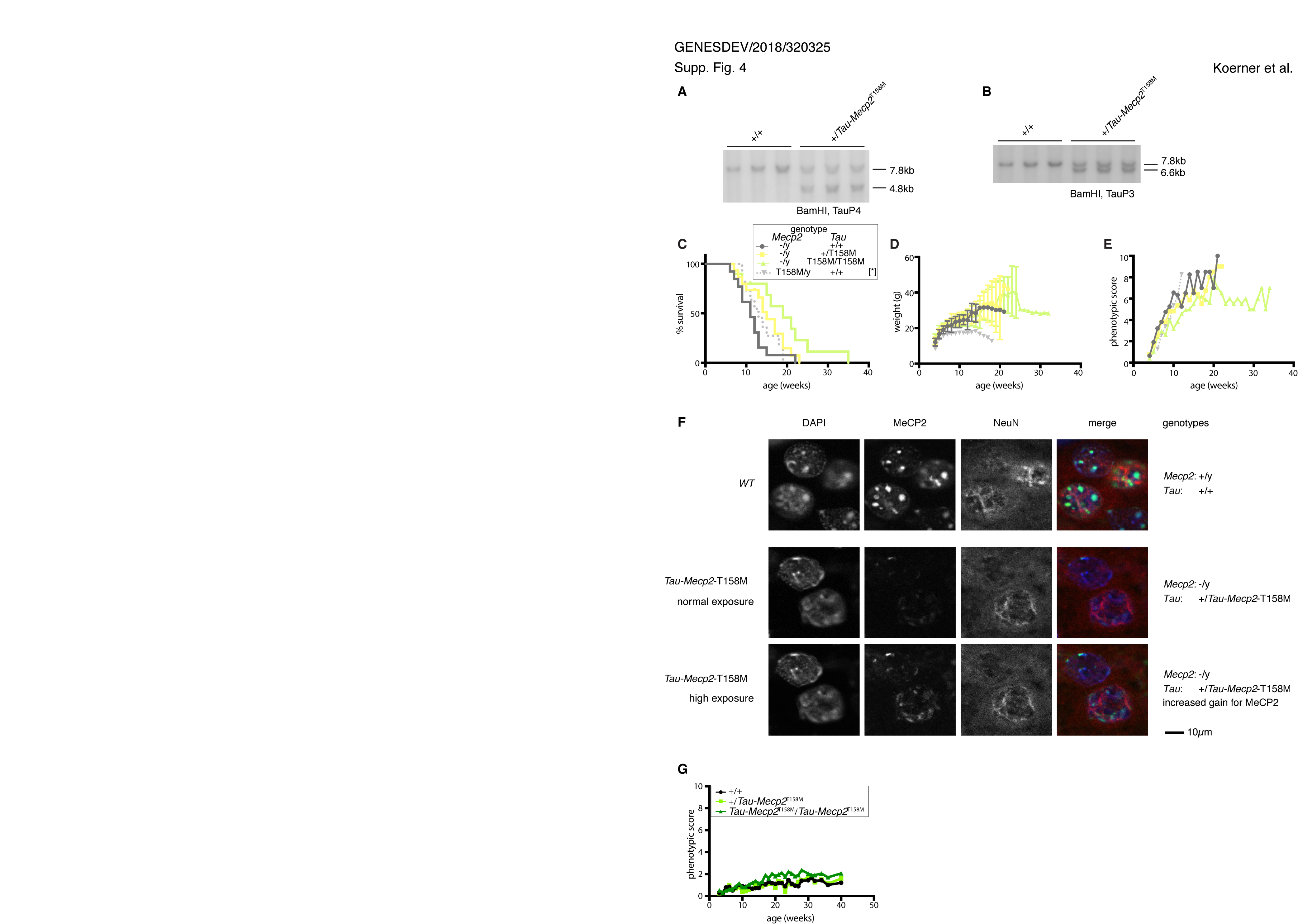
*

*Supp. Fig. 4: Generating Tau-MeCP2[T158M] mice.*

1. Southern blot of BamHI-digested tail DNA from *Tau-Mecp2*[T158M] mice and wildtype littermates using probe TauP4. For locus schematic see Supp. Fig. 1B.
2. Southern blot of BamHI-digested tail DNA of *Tau-Mecp2*[T158M] mice and wildtype littermates using probe TauP3.
3. Survival of a cohort comprising *Mecp2* -/y, +/+ (n=13, grey); *Mecp2* -/y, +/*Tau-Mecp2*[T158M] (n=15, yellow); *Mecp2* -/y, *Tau-Mecp2*[T158M]/*Tau-Mecp2*[T158M] (n=10, pale green). [*] As comparators, *Mecp2*[T158M]/y, *Tau* +/+ (n=11, light grey) are shown (Brown et al. 2016). Statistical analysis was performed using a Mantel-Cox test: *Mecp2* -/y vs *Mecp2* -/y, +/*Tau-Mecp2*[T158M]: p=0.0528 (ns). *Mecp2* -/y vs *Mecp2* -/y, *Tau-Mecp2*[T158M]/*Tau-Mecp2*[T158M]: p=0.0101 (*).
4. Body weight of the mice shown in C) (mean and standard deviation). Due to the high number of animals dying during the course of the study, a statistical analysis is not possible.
5. Phenotypic scores of the mice shown in C). Due to the high number of animals dying during the course of the study, a statistical analysis is not possible.
6. Immunofluorescence of the hippocampus CA3 brain section of male *Mecp2* -/y, *Tau* +/ *Tau-Mecp2* [T158M] mice (7 weeks). Wildtype (same image as in Fig. 1E) is shown for comparison. Sections were stained for DAPI, MeCP2 and NeuN. As expression of MeCP2[T158M] protein is low, two different exposures of the same nuclei in this channel are shown, to facilitate visualisation of subcellular MeCP2 localisation.
7. Compound phenotypic score of the mice shown in Fig. 4D. Two-way repeated measures ANOVA (week 3 and 40 excluded as not all animals were analysed, 1 WT and 2 het animals that died were excluded, too; WT n=8, het n=8, hom n=10). Genotype effect F(2,23)=3.155. p=0.0616 (ns).

*
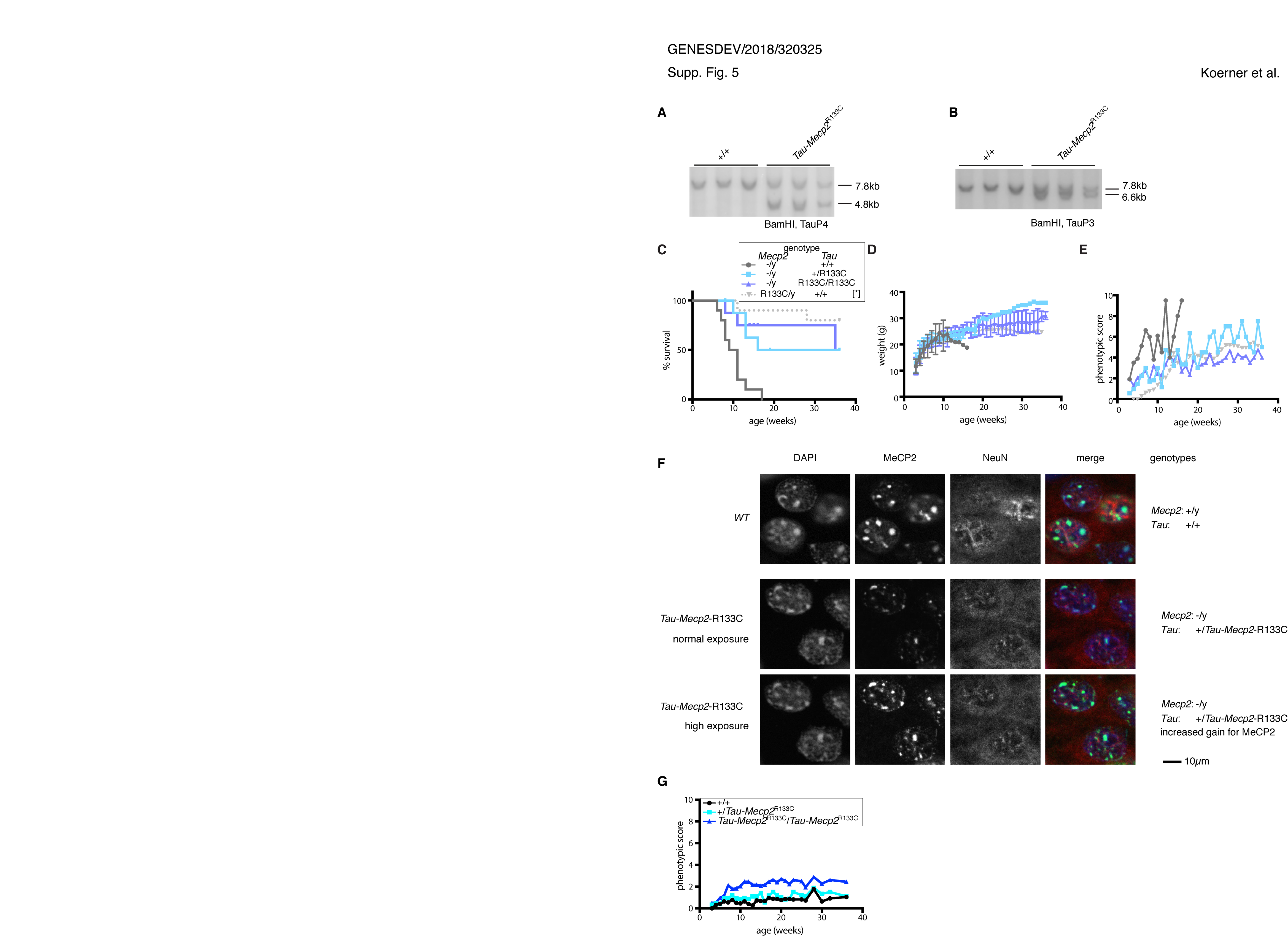
*

*Supp. Fig. 5: Generation of Tau-Mecp2[R133C] mice*

1. Southern blot of BamHI-digested tail DNA from *Tau-Mecp2*[R133C] mice and wildtype littermates using probe TauP4. For locus schematic see Supp. Fig. 1B.
2. Southern blot of BamHI-digested tail DNA from *Tau-Mecp2*[R133C] mice and wildtype littermates using probe TauP3.
3. Survival of a cohort comprising *Mecp2* -/y, +/+ (n=10, grey); *Mecp2* -/y, +/*Tau-Mecp2*[R133C] (n=10, pale blue); *Mecp2* -/y, *Tau-Mecp2*[R133C]/*Tau-Mecp2*[R133C] (n=8, dark blue). [*] As comparators, *Mecp2*[R133C] /y, *Tau* +/+ (n=10, light grey) are shown (Brown et al. 2016). This was analysed using a Mantel-Cox test: *Mecp2* -/y vs *Mecp2* -/y, +/*Tau-Mecp2*[R133C]: p=0.0027 (***); *Mecp2* -/y vs *Tau-Mecp2*[R133C]/*Tau-Mecp2*[R133C]: p=0.0024 (***); *Mecp2* -/y, +/ *Tau-Mecp2*[R133C] vs *Mecp2*[R133C]/*Tau-Mecp2*[R133C]: p=0.0024 (***).
4. Body weight of the mice shown in C) (mean and standard deviation). Due to the high number of animals dying during the course of the study, a statistical analysis is not possible.
5. Phenotypic score of the mice shown in C). Due to the high number of animals dying during the course of the study, a statistical analysis is not possible.
6. Immunofluorescence of the hippocampus CA3 brain section of *Mecp2* -/y, *Tau* +/ *Tau-Mecp2*[R133C] male mice (7 weeks). Wildtype (same image as in Fig. 1E) is shown for comparison. Sections were stained for DAPI, MeCP2 and NeuN. As expression of MeCP2[R133C] protein is low, two different exposures of the same nuclei are shown to facilitate visualisation of subcellular MeCP2 localisation.
7. Compound phenotypic score of the mice shown in Fig. 4J. Two-way repeated measures ANOVA (week 3 was excluded as not all animals were analysed; 1 het and 3 hom animals which died were excluded, too; WT n=11, het n=9, hom n=9). Genotype effect F(2,26)=16.82. P<0.0001 (****).


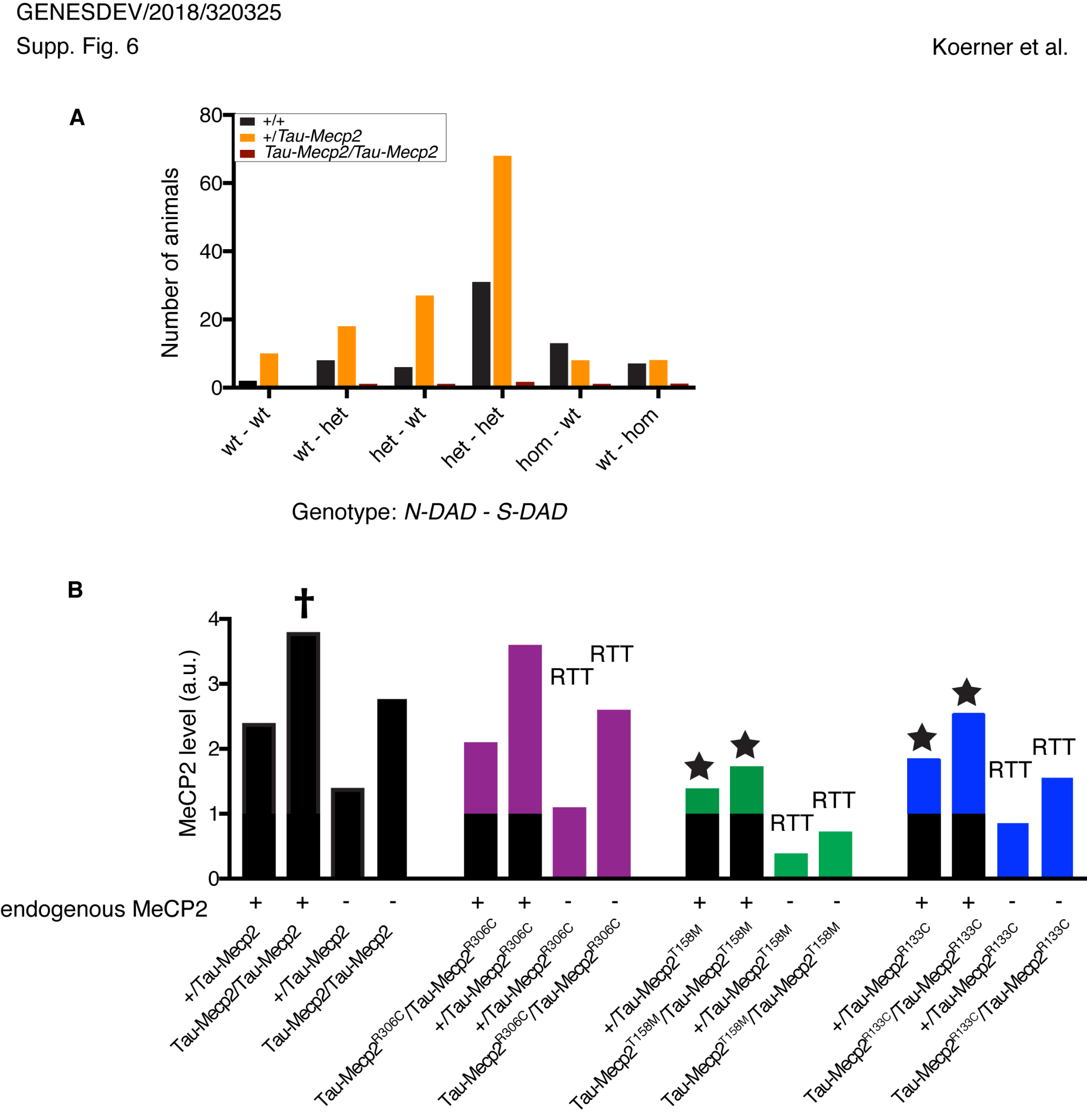


*Supp. Fig. 6: Reducing HDAC3 activity does not rescue Tau-MeCP2 lethality.*

1. Numbers of all animals surviving to weaning when wildtype, heterozygous or homozygous for *Tau-Mecp2* and wildtype, heterozygous or homozygous for N-DAD and S-DAD mutations. The figure extends Fig. 5D.
2. Graph displaying wildtype (black) and mutant (purple = R306C, green = T158M, blue = R133C) MeCP2 levels in the different lines analysed in this study. Protein levels for *Tau-MeCP2/Tau-MeCP2* have been extrapolated from values obtained for heterozygous animals. The cross indicates lethality, RTT indicates a Rett syndrome-like phenotype, and the star indicates a toxic effect manifested by hindlimb clasping.

**Supplemental Reference**

Brown K, Selfridge J, Lagger S, Connelly J, De Sousa D, Kerr A, Webb S, Guy J, Merusi C, Koerner MV et al. 2016. The molecular basis of variable phenotypic severity among common missense mutations causing Rett syndrome. *Hum Mol Genet* **25**: 558-570.
